# Star-like Cobalt Sulfide Nanoarrays Coupled with Fe Single-Atom Catalyst as Binder-Free Integrated Cathodes for Efficient and Robust Seawater Zinc–Air Batteries

**DOI:** 10.3390/molecules31122064

**Published:** 2026-06-12

**Authors:** Xuehan Zheng, Zhicheng Wang, Zhi Jiang, Haoxiong Nan, Junmin Luo, Chenghang You

**Affiliations:** 1 School of Chemistry and Chemical Engineering, Hainan University, Haikou 570228, China; 20233002963@hainanu.edu.cn; 2 Key Laboratory of Water Pollution Treatment and Resource Reuse of Hainan Province, The International Joint Research Center for Clean and Efficient Utilization of Hydrocarbon Resources in the South China Sea of Hainan Province, School of Chemistry and Chemical Engineering, Hainan Normal University, Haikou 571158, China; 202412070300019@hainnu.edu.cn (Z.W.); 202107080116@hainnu.edu.cn (Z.J.); 3 School of Marine Technology and Equipment, Hainan University, Haikou 570228, China; luojunming@hainanu.edu.cn

**Keywords:** seawater zinc–air battery, binder-free integrated cathode, single-atom catalyst, cobalt sulfide, bifunctional oxygen catalysts

## Abstract

Seawater zinc–air batteries (SZABs) stand out as promising candidates for marine and offshore energy supply. However, their practical implementation is greatly restricted by tardy oxygen reduction reaction (ORR) and oxygen evolution reaction (OER) kinetics at the air cathode, severe chloride ion-induced catalyst corrosion, and structural deterioration of traditional binder-containing electrodes in seawater media. Herein, we design and fabricate a binder-free integrated electrode consisting of carbon-supported iron phthalocyanine- modified star-like cobalt sulfide arrays directly grown on nickel foam. The optimal catalyst (0.3FePc-C/CoS) integrates the respective advantages of Fe single atoms and cobalt sulfide, exhibiting excellent ORR and OER activity, delivering a prominent half-wave potential of 0.89 V versus RHE, and exhibiting a low OER overpotential of 160 mV at 50 mA cm^−2^ and robust stability in seawater. As a self-supported air cathode, the 0.3FePc-C/CoS-based battery attains a favorable open-circuit voltage reaching 1.48 V, prominent peak power density (126.4 mW cm^−2^), small charge–discharge potential polarization (0.52 V), excellent energy efficiency (68.8%) and extraordinary long-term cycling durability (>360 h). This work not only discloses a feasible synergistic modulation strategy for constructing high-performance bifunctional electrocatalysts but also provides a valuable reference for developing corrosion-resistant integrated air electrodes toward practical marine energy storage applications.

## 1. Introduction

Fossil resource depletion and environmental degradation have prompted extensive research into electrochemical energy storage and conversion technology. By virtue of their great theoretical energy density (≈1218 Wh kg^−1^), abundant and low-cost zinc reserves, intrinsic safety, environmental friendliness, and unique capability of directly using natural seawater without desalination, rechargeable seawater zinc–air batteries (SZABs) are highly competitive candidates for marine and offshore applications [1,2,3,4,5,6,7,8] (e.g., island power supply, autonomous marine equipment, and buffering of intermittent ocean renewable energy output), where freshwater supply is limited, grid infrastructure is absent, and long-distance energy transport is prohibitively costly.

Nevertheless, the practical deployment of SZABs is profoundly impeded by multiple interrelated challenges [3]. First, the intrinsically sluggish multi-electron transfer kinetics of the oxygen reduction reaction (ORR) and the oxygen evolution reaction (OER) result in large overpotentials, poor rate capability, and low energy efficiency. Second, the Cl^−^ in seawater competes with OH^−^ for adsorption on active sites and induces catalyst corrosion [9,10,11,12], leading to progressive performance degradation; meanwhile, traditional binder-containing electrodes suffer from blocked active sites, increased interfacial charge-transfer resistance, and structural delamination under prolonged immersion, further compromising output performance and service life.

Among these challenges, the OER performance and stability of the air electrode under seawater conditions attract particular attention. Usually, OER proceeds at substantially higher anodic potentials (typically >2.0 V in practical devices) than ORR, driving the oxidation and dissolution of transition metallic catalytic sites. In seawater, this degradation is further exacerbated by the synergistic action of high oxidation potentials and aggressive Cl^−^ attack, resulting in pitting corrosion and accelerating catalyst dissolution. Consequently, the advancement of bifunctional catalysts with superior inherent OER catalytic performance, alongside strong immunity to Cl^−^ corrosion, represents a critical yet unmet need for achieving high-efficiency and long-life rechargeable SZABs.

Currently, advanced bifunctional catalysts rely heavily on noble metals [13,14,15,16,17,18,19,20], which exhibit respectable activity but suffer from scarcity, prohibitive cost, and poor bifunctional stability under alternating ORR/OER potentials. Cobalt sulfides are promising OER catalysts for seawater splitting due to their high OER activity and the Cl^−^ repelling ability originating from the in situ formed sulfate layer during the OER [21,22,23,24]. However, pristine cobalt sulfide exhibits intrinsically poor ORR activity. In stark contrast, iron single-atom catalysts (Fe SACs) are active for ORR but inactive for OER and suffer from instability due to rapid oxidative degradation under the highly oxidizing potentials required for OER (full-cell charging voltages usually >2.0 V) [25,26,27], leading to demetallation and structural fragmentation. Rationally integrating these two candidate catalysts should potentially compensate for their respective deficiencies and achieve favorable bifunctional ORR/OER activity [28]. Besides compositions, bifunctional catalysts’ performance also depends strongly on electrode structures. Conventional powder catalysts require polymeric binders and conductive additives to fabricate electrodes, which introduce multiple detrimental effects, e.g., burial of active sites, impeded reactant/product mass transport, high interfacial charge-transfer resistance, and mechanical delamination under cyclic gas evolution/consumption. In contrast, binder-free integrated electrodes fabricated by growing active materials on three-dimensional conductive substrates eliminate these drawbacks while providing continuous electron transport pathways, open porous architectures for rapid electrolyte permeation and gas diffusion, and enhanced mechanical stability, features imperative for long-term operation in corrosive seawater.

Motivated by the above considerations, herein, we report a series of integrated nickel foam (NF) based binder-free air electrodes via a facile strategy comprising solution immersion and hydrothermal reaction. The optimized catalyst (0.3FePc-C/CoS) exhibits superior bifunctional activity and outstanding stability. As a self-supported air cathode, it delivers an SZAB a high open-circuit voltage of 1.48 V, remarkable peak power density (126.4 mW cm^−2^), and ultra-high cycling stability (>360 h). Specifically, energy efficiency reaches 68.8%, significantly outperforming other recently reported zinc–air batteries (ZABs). The insights gained from this study are expected to provide generalizable principles for developing active and robust integrated air electrodes for practical large-scale marine energy storage applications.

## 2. Results

### 2.1. Physicochemical Characterization

**Figure 1:**
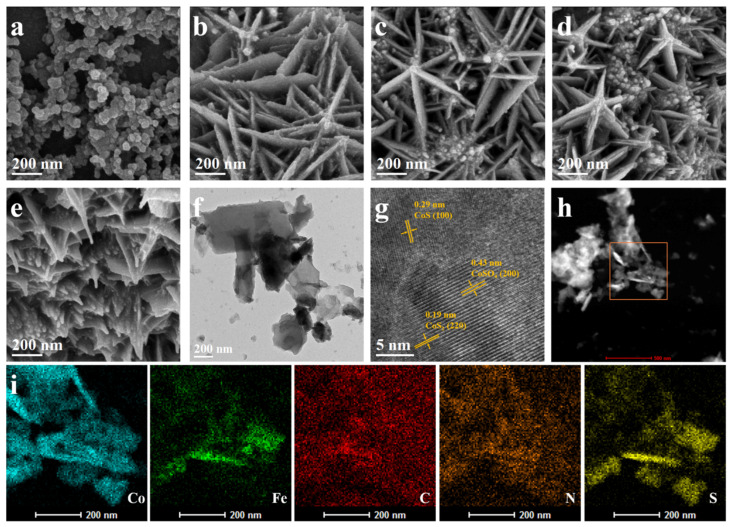
SEM images: (**a**) FePc-C; (**b**) CoS; (**c**) 0.1FePc-C/CoS; (**d**) 0.3FePc-C/CoS; (**e**) 0.5FePc-C/CoS; (**f**) TEM image of 0.3FePc-C/CoS; (**g**) HRTEM image of 0.3FePc-C/CoS; (**h**) STEM image of 0.3FePc-C/CoS and the orange box indicates the region of EDX Mapping.; (**i**) EDX Mapping images of 0.3FePc-C/CoS.

The structural configuration and microscopic morphology of the as-prepared catalysts were explored by SEM. The SEM image of pure FePc-C (Figure 1a) shows aggregated nanoparticles with sizes of ~20–50 nm, indicating a typical carbon-based nanomaterial morphology with an inherent tendency to agglomerate. For sole CoS (Figure 1b), a well-defined array structure composed of numerous aligned nanosheets is observed.

Figure 1c–e reveals the morphologies of the hybrid catalysts with different FePc-C contents. When FePc-C loading is low (0.1FePc-C/CoS, Figure 1c), the CoS nanosheet array is largely preserved, with only a small number of FePc-C nanoparticles sparsely distributed on the surface. When the FePc-C content increases to 0.3 g (0.3FePc-C/CoS, Figure 1d), FePc-C nanoparticles are uniformly and densely decorated among the CoS nanosheets without obvious agglomeration, while the intercrossed nanosheet array structure of CoS remains intact. However, when excessive FePc-C was added, severe aggregation of nanoparticles occurred on the CoS surface (0.5FePc-C/CoS, Figure 1e), which is supposed to block the active sites and hinder mass transfer, thus potentially deteriorating the electrocatalytic performance.

**Figure 2:**
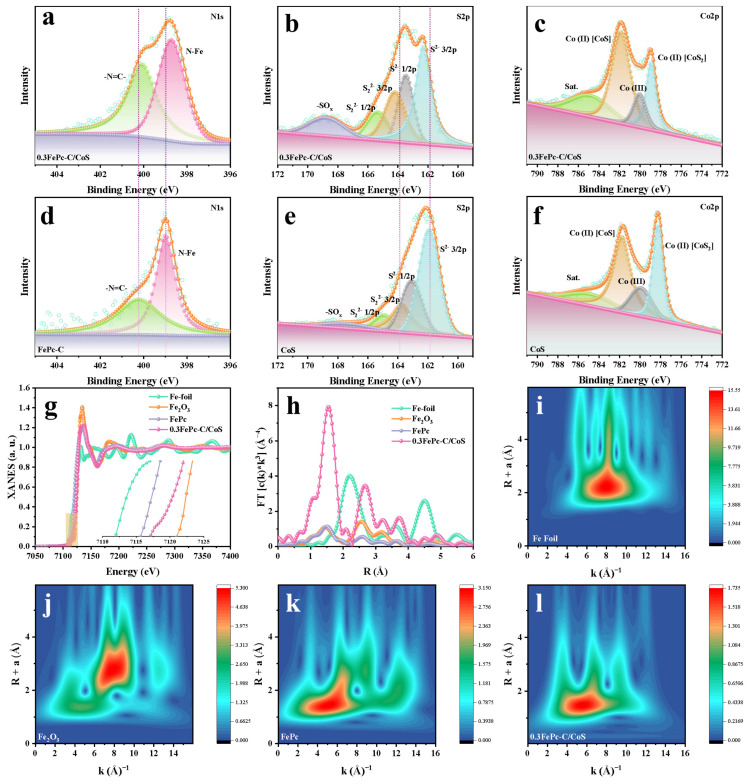
(**a**) N1s spectra of 0.3FePc-C/CoS; (**b**) S2p spectra of 0.3FePc-C/CoS; (**c**) Co2p spectra of 0.3FePc-C/CoS; (**d**) N1s spectra of FePc-C; (**e**) S2p spectra of CoS; (**f**) Co2p spectra of CoS; (**g**) XANES of 0.3FePc-C/CoS; (**h**) R space; WT-EXAFS of (**i**) Fe Foil; (**j**) Fe_2_O_3_; (**k**) FePc; (**l**) 0.3FePc-C/CoS.

The TEM image (Figure 1f) further confirms the coexistence of thin CoS nanosheets and FePc-C nanoparticles anchored on their surface. HRTEM (Figure 1g) reveals distinguishable crystalline fringes featuring interplanar distances of 0.29, 0.19, and 0.45 nm, assigned to the (100), (220), and (200) planes of CoS, CoS_2_, and CoSO_4_, respectively, indicating the coexistence of CoS and CoS_2_ in 0.3FePc-C/CoS. The presence of the CoSO_4_ phase should originate from the partial oxidation of the catalyst in air, which can be further proved by the sulfate-related peaks in its S 2p XPS spectra (Figure 2b). STEM (Figure 1h) and EDX mapping were further performed to analyze the elemental distribution of 0.3FePc-C/CoS. The corresponding EDX elemental mapping (Figure 1i) reveals the uniform dispersion of Co, S, N, Fe, and C throughout the 0.3FePc-C/CoS, confirming the successful integration of FePc-C and CoS.

The surface compositions and electronic properties of the as-synthesized catalysts were characterized via XPS. For the N 1s spectrum of FePc-C (Figure 2d), two characteristic peaks are observed at ~398.5 and ~400.0 eV, in accordance with Fe-N coordination (pyrrolic N in FeN4) and aza-bridging N (meso-N) in the phthalocyanine macrocycle, respectively, confirming the intact FeN_4_ coordination structure. After integration with CoS to form 0.3FePc-C/CoS (Figure 2a), the N 1s peaks exhibit a slight red shift of ~0.2 eV, revealing a higher electron density surrounding Fe-N4 moieties.

The S 2p spectrum of sole CoS (Figure 2e) shows three main components: the S^2−^ 3/2p (~161.9 eV) and S^2−^ 1/2p (~163.1 eV) peaks originating from metal sulfides, a minor S_2_^2−^ doublet (~163.8 and ~165.0 eV) attributable to trace surface disulfide species, and a small -SOx peak (~167.3 eV) corresponding to surface oxidized sulfur. For 0.3FePc-C/CoS (Figure 2b), the S_2_^2−^ peaks are more pronounced compared to pure CoS, suggesting FePc-C can promote the formation of CoS_2_. Moreover, both the S^2−^ and S_2_^2−^ peaks exhibit a blue shift of ~0.4 eV, indicating that the electron density around sulfur atoms decreased, as well as their participation in interfacial charge transfer.

The Co 2p profile of the CoS (Figure 2f) exhibits typical Co 2p3/2 diffraction peaks corresponding to Co(II) in the sulfide matrix (~778.8 and ~781.7 eV) and surface oxidized Co species (~780.0 eV), along with characteristic satellite peaks. For 0.3FePc-C/CoS (Figure 2c), the Co 2p profile exhibits differential shifts for the two cobalt sulfide phases: the Co(II) species in CoS show a modest blue shift of ~0.2 eV, indicating mild electronic perturbation; in contrast, the Co(II) species in CoS_2_ exhibit a more pronounced blue shift of ~0.7 eV, suggesting substantial electron density reduction and stronger interfacial coupling. These complementary observations provide spectroscopic evidence of the interfacial electron transfer from both CoS and CoS_2_ to FePc-C, with CoS_2_ serving as the more active electron donor.

To probe the first-shell coordination sphere of Fe, XANES and EXAFS were performed. Figure 2g illustrates that the Fe K-edge absorption edge energy for 0.3FePc-C/CoS is more positive than that of FePc but lower than that of Fe_2_O_3_, indicating that the Fe in the catalyst has an oxidation state ranging from +2 to +3. The FT-EXAFS spectra of 0.3FePc-C/CoS (Figure 2h) show a dominant peak attributed to Fe-N scattering (~1.50 Å), which can also be confirmed by their similar peak intensities at approximately 4–6 Å-1 in its wavelet transform (WT) analysis of EXAFS illustrated in Figure 2i–l. No Fe-Fe, Fe-Co or Fe-O scattering signals are detected in both R space and WT-EXAFS images, which further verifies the atomic dispersion of Fe, as well as the absence of Fe-O and Fe-Co bonds in it. Also, the absence of Fe-Co bonds demonstrates that cobalt sulfide is only in intimate contact with the carbon framework of FePc-C.

### 2.2. Electrochemical Characterization

**Figure 3:**
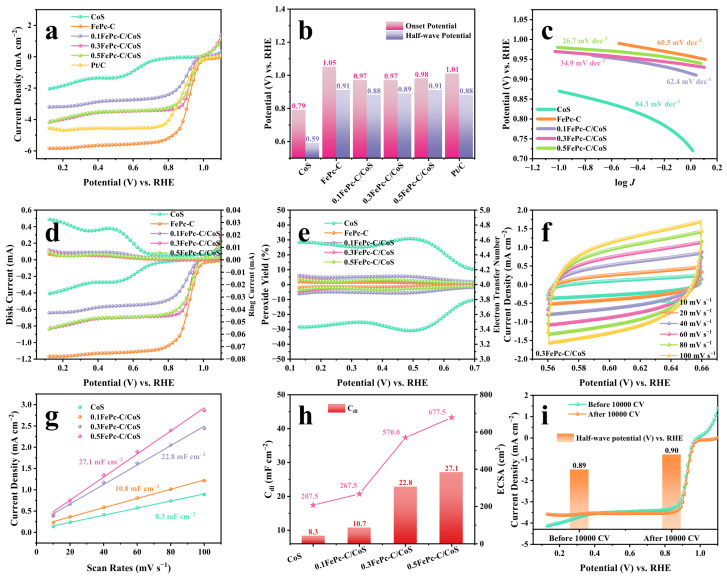
(**a**) ORR polarization profiles of obtained catalysts; (**b**) half-wave potentials of different catalysts; (**c**) Tafel analysis based on the LSV curves; (**d**) RRDE measurement results; (**e**) peroxide (H_2_O_2_) yields and electron transfer numbers based on the RRDE measurement results; (**f**) CV curves of 0.3FePc-C/CoS; (**g**) Cdl; (**h**) Cdl and ECSA values of various catalysts; (**i**) LSV curve before and after 10,000 CV cycles.

Figure 3a shows the ORR polarization curves of different catalysts. The pristine CoS sample shows inferior ORR capability, reaching a half-wave potential (E_1/2_) of merely 0.67 V, while FePc-C shows significantly enhanced performance (E_1/2_ = 0.91 V). After introducing FePc-C into CoS, the catalysts’ performance was significantly enhanced. When the dosage of FePc-C was increased to 0.5 g, the E_1/2_ of the catalyst (0.5FePc-C/CoS) was equivalent to that of FePc-C (Figure 3b), implying that the ORR performance mainly originates from the FePc-C component. Tafel curves (Figure 3c) show that the composite catalysts exhibit lower Tafel slopes, suggesting an improved kinetic process of ORR on the catalyst. Among them, 0.3FePc-C/CoS had the lowest Tafel slope (26.7 mV·dec^−1^), much smaller than those of CoS (84.3 mV·dec^−1^) and 0.1FePc-C/CoS (34.9 mV·dec^−1^), confirming the faster ORR kinetic process on 0.3FePc-C/CoS. As for rotating ring-disk electrode (RRDE) tests (Figure 3d,e), CoS had the lowest electron transfer number (*n* < 3.5) and the highest H_2_O_2_ yield (>30%). In contrast, the catalysts containing FePc-C exhibited much higher electron transfer numbers and lower peroxide yields (<5%), indicating that these catalysts have high selectivity for the efficient four-electron pathway. The double-layer capacitance (C_dl_) and electrochemically active surface area (ECSA) measured from cyclic voltammetry (CV) curves (Figure 3f and Figures S1–S3) at different scan rates (Figure 3f–h) show that 0.5FePc-C/CoS had the highest Cdl of 27.1 mF cm^−2^ and the largest ECSA of 677.5 cm^−2^, which are remarkably superior to those of bare CoS (8.3 mF·cm^−2^, 207.5 cm^−2^) and 0.1FePc-C/CoS (10.7 mF·cm^−2^, 267.5 cm^2^), suggesting that FePc-C can drastically enhance catalysts’ ECSA. Figure 3i shows the ORR polarization curves of 0.3FePc-C/CoS before and after 10,000 CV cycles. The extra current increase in the fresh sample (green curve) at low potentials can be attributed to side reactions such as hydrogen evolution. After accelerated aging, these parasitic reactions are suppressed, resulting in a smooth limiting current platform for the aged catalyst. The limiting diffusion current platforms before and after aging nearly overlap, and the half-wave potential positively shifts from 0.89 V to 0.90 V vs. RHE, implying that the catalytic activity is not degraded but slightly improved after durability cycling, confirming the outstanding stability of the as-prepared catalyst.

**Figure 4:**
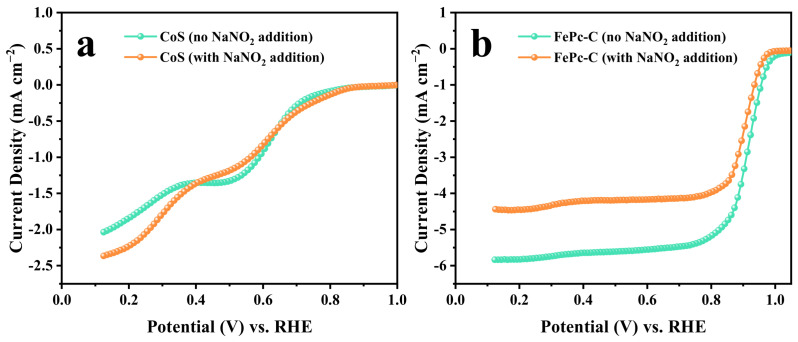
Selective poisoning for different catalysts: (**a**) CoS; (**b**) FePc-C.

To identify the true active site for ORR in our catalysts, selective nitrite poisoning control experiments on bare CoS and FePc-C were carried out, and corresponding LSV results are displayed in Figure 4.

According to electrochemical data, the introduction of sodium nitrite barely alters the ORR activity of pristine CoS (Figure 4a), whereas obvious decay of ORR catalytic performance is observed for FePc-C after nitrite addition (Figure 4b). This result demonstrates that Fe single-atom sites dominate the overall ORR activity of the composite catalyst, and no synergistic catalytic effect between Fe and Co sites occurs in our system.

**Figure 5:**
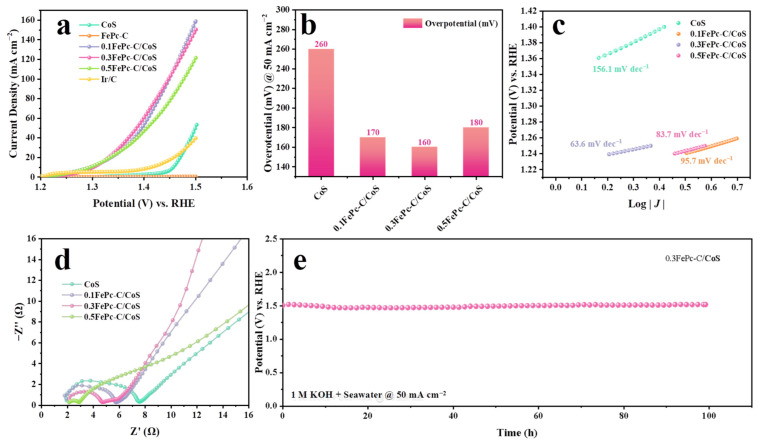
(**a**) OER LSV curves; (**b**) overpotentials of different catalysts; (**c**) Tafel analysis based on the LSV curves; (**d**) EIS measurements; (**e**) P-t curve of 0.3FePc-C/CoS.

Figure 5a shows the OER polarization curves of each catalyst. FePc-C exhibits negligible OER activity, while pure CoS shows moderate activity. Notably, the 0.3FePc-C/CoS hybrid catalyst displays the optimal OER performance. As shown in Figure 5b, the overpotential of 0.3FePc-C/CoS at 50 mA·cm^−2^ is only 160 mV, which is significantly lower than that of CoS (260 mV), 0.1FePc-C/CoS (170 mV) and 0.5FePc-C/CoS (180 mV). These results suggest that the OER performance mainly comes from the CoS and indicate that proper addition of FePc-C can effectively enhance OER activity. The decreasing OER performance of 0.5FePc-C/CoS should originate from the covering of cobalt sulfide sites by excess FePc-C introduction (Figure 1d). The reaction kinetics were further investigated by Tafel analysis (Figure 5c). The 0.3FePc-C/CoS hybrid delivers a Tafel slope of 63.6 mV·dec^−1^, obviously lower than 156.1 mV·dec^−1^ for pure CoS and 95.7 mV·dec^−1^ for 0.1FePc-C/CoS. As for EIS (Figure 5d), as the FePc-C content increases, the diameter of the high-frequency semicircle gradually decreases, indicating that FePc-C can remarkably improve catalysts’ electrical conductivity. And this improved electrical conductivity, we suggest, should be responsible for the enhanced OER performance of FePc-C-containing catalysts. The degraded OER performance of 0.5FePc-C/CoS should also be attributed to the excessive FePc-C that shields the OER-active cobalt sulfide sites. Figure 5e presents the potential-time (P-t) curve of 0.3FePc-C/CoS at 50 mA cm^−2^. It can be observed that no obvious potential rise was detected after 100 h of operation, demonstrating the outstanding OER stability of the 0.3FePc-C/CoS.

### 2.3. Battery Characterization

**Figure 6:**
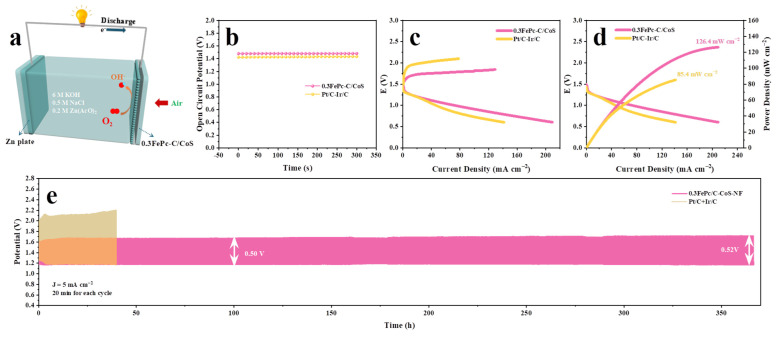
(**a**) homemade SZAB; (**b**) OCPs of SZABs using 0.3FePc-C/CoS and Pt/C-Ir/C catalysts, respectively; (**c**) charging–discharging curves of the two SZABs; (**d**) discharging curves and power densities of the two fabricated SZABs; (**e**) cyclic charging–discharging curves of the SZABs.

To delve deeper into the practical application prospect of the 0.3FePc-C/CoS catalyst, a homemade SZAB was fabricated (Figure 6a). The 0.3FePc-C/CoS-based SZAB delivers a high open-circuit potential (OCP) of 1.48 V, outperforming the counterpart assembled with Pt/C-Ir/C (Figure 6b). In terms of charge–discharge polarization curves (Figure 6c), the SZAB equipped with 0.3FePc-C/CoS presents a smaller potential deviation between galvanostatic cycling profiles at diverse current densities, confirming the excellent bifunctional catalytic activity of 0.3FePc-C/CoS. Discharge profiles and the matched power density curves (Figure 6d) reveal that the SZAB with 0.3FePc-C/CoS delivers an ultimate power density of 126.4 mW cm^−2^, far exceeding the 85.4 mW cm^−2^ recorded for the Pt/C-Ir/C-based device. During the long-term cycling stability measurements (Figure 6e), the 0.3FePc-C/CoS-based SZAB maintains stable charge–discharge performance over 360 h, along with the potential difference only slightly rising from 0.50 V to 0.52 V. By comparison, the Pt/C-Ir/C assembled SZAB shows a remarkable voltage gap widening and severe performance degradation after only tens of hours of cycling. The superior stability of the 0.3FePc-C/CoS-based SZAB originates from the robust interfacial coupling between FePc-C and CoS, which suppresses the over-oxidation of active sites during OER, and the binder-free integrated structure, which ensures structural integrity during long-term cycling. As for energy efficiency, the 0.3FePc-C/CoS-based SZAB exhibits an energy efficiency of 68.8%, obviously higher than that of the ones using Pt/C-Ir/C catalysts. So far as we know, it also surpasses other SZABs recently reported (Table S1).

## 3. Materials and Methods

### 3.1. Materials

DMF, iron phthalocyanine, cobalt chloride and potassium hydroxide (KOH) were acquired from Macklin Co., Ltd. (Shanghai, China). Gas diffusion layer (GDL, Sigracet-28BC) was supplied by SGL Carbon SE (Wiesbaden, Germany). Carbon powder (Ketjenblack EC-300J, ~800 m^2^g^−1^) was purchased from Lion Specialty Chemicals Co., Ltd. (Tokyo, Japan). All raw reagents were of analytical purity grade and were employed straightaway without any further purification or preprocessing.

The nickel foam (NF, purity > 99.9%) used in this work was purchased from Hefei Kejing Materials Technology Co., Ltd. (Hefei, China). The thickness of the NF is 1.6 mm, with an areal density of 346 g/m^2^ and porosity ≥95%. The integrated cathode was cut into the required sizes (1 × 2 cm) from the original NF sheet before use.

Prior to loading catalysts, the NF substrate was pre-cleaned via sequential ultrasonication: 2 M HCl aqueous solution for 15 min (to eliminate surface NiO passivation layer), anhydrous ethanol for 10 min (to remove organic grease), and deionized water for three cycles (5 min per cycle). After washing, the cleaned NF was dried at 60 °C overnight in an oven for subsequent electrode fabrication.

### 3.2. Catalysts Fabrication

**Figure 7:**
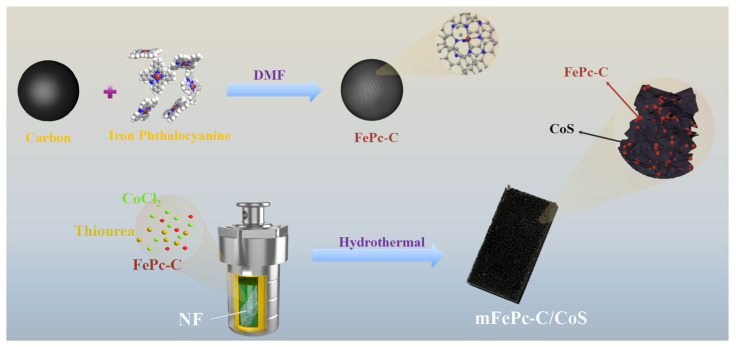
Preparation of the binder-free integrated electrode.

Iron phthalocyanine (0.3 g) was dispersed in 100 mL DMF under ultrasonication. Then, 1.0 g of carbon powder was added (Figure 7), and the mixture was magnetically stirred for 2 h before it was centrifuged. The obtained solid was then thoroughly rinsed with ethanol to remove residual DMF and unadsorbed FePc and dried to yield the FePc-C composite (yield: ~87.4%).

Next, 0.3 g FePc-C and 4.0 g thiourea were dispersed in 60 mL 0.1 M aqueous CoCl_2_ solution under ultrasonication. The suspension was then sealed in an autoclave after the addition of a cleaned nickel foam (NF) substrate and kept at 120 °C for a duration of 4 h. Upon spontaneous cooling, the integrated electrode was obtained, which was then sequentially washed using deionized water and ethanol, then dried under vacuum. The resulting binder-free electrode is denoted as 0.3FePc-C/CoS, where the number represents the mass (in g) of FePc-C used in the synthesis. Control samples, including 0.1FePc-C/CoS, 0.5FePc-C/CoS, and pure CoS (without FePc-C), were prepared via an identical procedure by adjusting the FePc-C dosage accordingly.

### 3.3. Working Electrode Fabrication

In all electrochemical measurements, a 5 mm diameter glassy carbon electrode (GCE) was adopted. Prior to every test, the vitreous carbon electrode was initially ultrasonically treated in ethanol, then polished using 50 nm α-Al_2_O_3_ powder suspension until its surface became smooth and mirror-like, and finally fully washed with deionized water.

Next, a 5.0 mg stripped catalyst sample was blended into 1 mL 0.25 wt% Nafion-ethanol solution and ultrasonicated for half an hour to obtain a uniform slurry. A volume of 20 μL ink was dripped onto the polished GCE and air-dried spontaneously, achieving a catalyst loading of 0.5 mg cm^−2^. Commercial 20% Pt/C was measured under the same parameters as the reference sample for ORR performance contrast.

For OER tests, the integrated electrodes were adopted directly without further treatment.

### 3.4. Air Electrode Fabrication

The optimized 0.3FePc-C/CoS integrated electrode was directly utilized as a cathode for seawater zinc–air battery (SZAB) tests, coupled with a blank SG-28BC gas diffusion layer (GDL, SIGRACET, Wiesbaden, Germany).

For benchmark comparison, a conventional air electrode was fabricated using a mixed catalyst of Pt/C and Ir/C. A catalyst ink containing 5.0 mg Pt/C and 5.0 mg Ir/C was prepared using the same procedure mentioned above, then coated over the GDL and dried via infrared irradiation.

### 3.5. Structural Characterizations

The microscopic morphology was characterized via JSM-7100F SEM (JEOL Ltd., Akishima, Tokyo, Japan) at 5 kV. JEM-2100 TEM was utilized at 200 kV to acquire fine structural information. XPS analyses were carried out on an ESCALAB 250 photoelectron spectrometer manufactured by Thermo VG Scientific (Manchester, UK). The nitrogen adsorption–desorption tests were performed using a TriStar II 3020 specific surface area tester. Moreover, XANES and EXAFS spectral data were obtained at the Shanghai Synchrotron Radiation Facility (Shanghai, China).

### 3.6. Electrochemical Analyses

All electrochemical measurements were implemented at ambient temperature via a CHI760E electrochemical analyzer in a conventional three-electrode system, which was further combined with a PINE rotating ring-disk electrode device. Herein, a mercury oxide electrode was selected as the reference electrode, alongside a platinum wire used as the counter electrode. All recorded potentials were converted into the reversible hydrogen electrode scale based on the Nernst formula:

E_(RHE)_ = E_(Hg/HgO)_ + 0.0592 × pH + 0.098

OER overpotential (η) was calculated as follows:

η = E_(RHE)_ − 1.23 V

For ORR tests, the electrolyte (0.1 M KOH + 0.5 M NaCl) was purged with oxygen over 30 min. Current densities were calculated based on the geometric area of the GCE (0.1964 cm^2^). For OER tests, 1.0 M KOH containing 0.5 M NaCl was used as the electrolyte.

LSV tests were conducted with a scanning speed of 5 mV per second. EIS were collected under open-circuit potential conditions, adopting an AC signal with 10 mV amplitude, covering frequencies between 10^5^ Hz and 0.01 Hz. The ECSA was quantified by means of C_DL_ measurement. The corresponding capacitance data were acquired from CV recorded at diverse scanning rates via [3,29,30]:

J = υ × C_DL_

in which J represents current density and υ stands for scanning speed.

ECSA was then calculated as:

ECSA = C_DL_/C_s_   (C_s_ = 0.040 mF cm^−2^).

RRDE tests were performed under the same conditions as LSV, with a ring potential of 0.5 V. Subsequently, the hydrogen peroxide production efficiency and electron transfer number (n) were determined using the following equations [20,29]:

n = 4I_d_/(I_d_ + I_r_/N)

H_2_O_2_ yield (%) = 200 × (I_r_/N)/(I_d_ + I_r_/N)

In the equation, I_d_ denotes disk current, I_r_ is ring current, and N is collection efficiency (0.36).

### 3.7. Zinc–Air Battery Measurements

Homemade SZABs were assembled for performance evaluation using a 6 M potassium hydroxide solution mixed with 0.5 M sodium chloride and 0.2 M Zn(Ac)_2_ as the electrolyte; a polished zinc foil was used as the anode. Charge–discharge polarization curves were recorded on a CHI760E workstation. Long-cycle charge–discharge tests were performed on a CT3004A battery tester (LANHE, Wuhan, China) at 5 mA cm^−2^. Each cycle comprised 10 min of discharging followed by 10 min of charging.

The energy efficiency (EE) of SZABs was calculated from the discharge and charge capacities [31,32]:

EE = (Q_discharge_/Q_charge_) × 100%

where Q_discharge_ and Q_charge_ represent the discharge and charge capacities obtained by integrating the current–time curves, respectively.

## 4. Conclusions

In summary, we successfully fabricated a binder-free integrated electrode consisting of carbon-supported iron phthalocyanine-modified star-like cobalt sulfide arrays directly grown on nickel foam for rechargeable seawater zinc–air batteries (SZABs). Benefiting from the strong synergistic catalytic effect, regulated electronic configuration, and the structural superiority of the binder-free integrated architecture, the optimal catalyst exhibits outstanding catalytic and robust stability in harsh corrosive seawater media. When employed as a self-supported air cathode, the assembled SZAB delivers a high open-circuit potential of 1.48 V and a prominent peak power density of 126.4 mW cm^−2^, showing a low-voltage polarization of 0.52 V during cycling, an impressively high energy efficiency of 68.8%, and an ultra-long cycling lifespan over 360 h, exhibiting remarkably competitive performance compared with the commercial Pt/C-Ir/C counterpart. The present work offers a feasible route to realize synergistic modulation for constructing high-efficiency bifunctional electrocatalysts and valuable research references for the rational design and development of corrosion-resistant integrated air cathodes. It is expected to promote the practical application of advanced seawater-based energy storage devices in marine and offshore energy supply systems.

## Data Availability

The original contributions presented in this study are included in the article/Supplementary Materials. Further inquiries can be directed to the corresponding authors.

## Supplementary Materials

The following supporting information can be downloaded at: https://www.mdpi.com/article/10.3390/molecules31122064/s1, Figure S1: CV curves of CoS under different scanning rates; Figure S2: CV curves of 0.1FePc-C/CoS under different scanning rates; Figure S3: CV curves of 0.5FePc-C/CoS under different scanning rates; Table S1: Performance comparison between the as-prepared 0.3FePc-C/CoS and the reported materials for ZABs [33,34,35,36,37,38,39,40,41,42,43,44,45,46,47].
