# Supplementary material for: Star-like Cobalt Sulfide Nanoarrays Coupled with Fe Single-Atom Catalyst as Binder-Free Integrated Cathodes for Efficient and Robust Seawater Zinc–Air Batteries
Source: Molecules. 2026 Jun 12;31(12):2064. doi: 10.3390/molecules31122064 (PMC13304826; doi:10.3390/molecules31122064)
Supplement: Supplementary file 1 [file molecules-31-02064-s001.zip › molecules-4352195-supplementary.pdf]

# Supplementary Files

# Star-like Cobalt Sulfide Nanoarrays Coupled with Fe Single-Atom Catalyst as Binder-Free Integrated Cathodes for Efficient and Robust Seawater Zinc–Air Batteries

**Xuehan Zheng [1], Zhicheng Wang [2], Zhi Jiang [2], Haoxiong Nan [1,*], Junmin Luo [3] and Chenghang You [2,*]**

[1] School of Chemistry and Chemical Engineering, Hainan University, Haikou 570228, China; 20233002963@hainanu.edu.cn

[2] Key Laboratory of Water Pollution Treatment and Resource Reuse of Hainan Province, The International Joint Research Center for Clean and Efficient Utilization of Hydrocarbon Resources in the South China Sea of Hainan Province, School of Chemistry and Chemical Engineering, Hainan Normal University, Haikou 571158, China; 202412070300019@hainnu.edu.cn (Z.W.); 202107080116@hainnu.edu.cn (Z.J.)

[3] School of Marine Technology and Equipment, Hainan University, Haikou 570228, China; luojunming@hainanu.edu.cn

* Correspondence: nanhaoxiong@hainanu.edu.cn (H.N.); you.ch@hainnu.edu.cn (C.Y.)

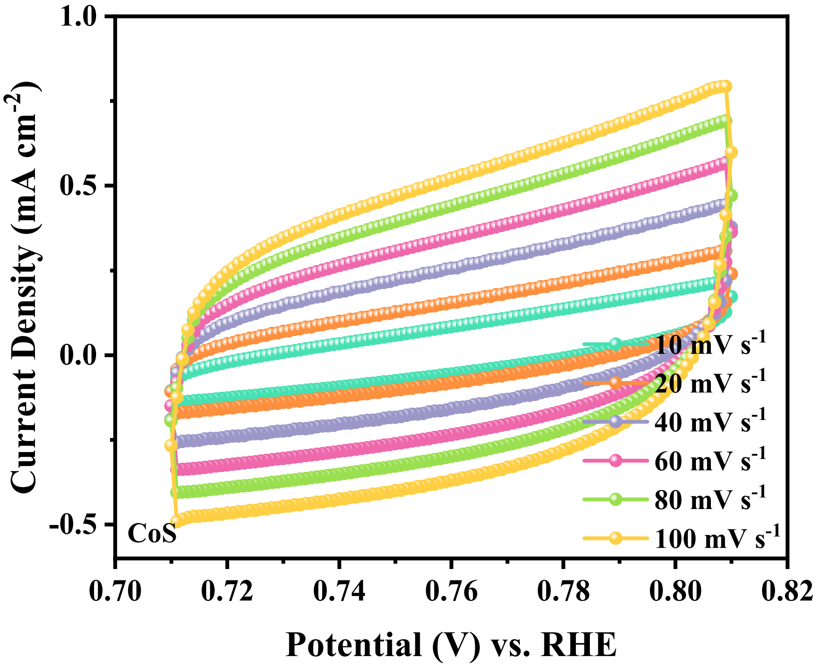


Fig. S1 CV curves of CoS under different scanning rates

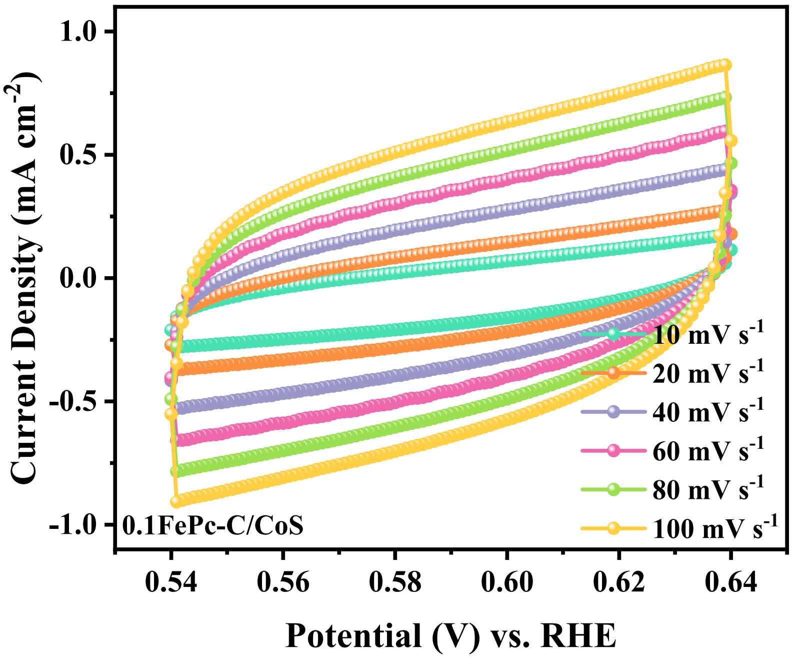


Fig. S2 CV curves of 0.1FePc-C/CoS under different scanning rates

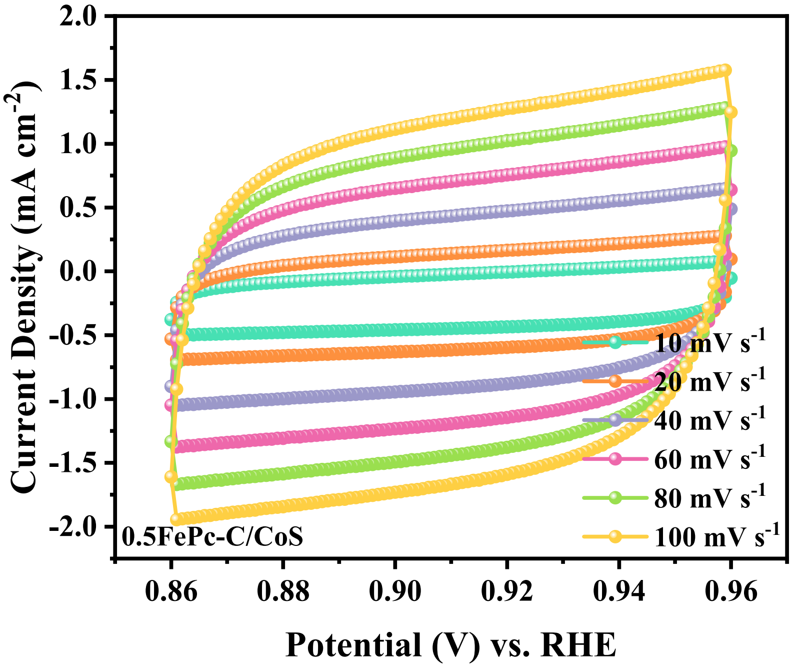


Fig. S3 CV curves of 0.5FePc-C/CoS under different scanning rates

**Table S1.** Performance comparison between the as-prepared 0.3FePc-C/CoS and the reported materials for ZABs.

| N | | Electrolyte | Potential gap between charging and discharging | Faradaic efficiency (%) | References |
|---|---|---|---|---|---|
| | **0.3FePc-C/CoS** | 0.5 M NaCl/6 M KOH/0.2 M $Zn(OAc)_2$ | **0.52** | **68.8** | **Our work** |
| 1 | BODIPY-Mn-bipy-I | 6 M KOH/0.2 M $Zn(OAc)_2$ | 0.99 | 54.4 | [1] |
| 2 | $Mo_2C$-$Fe_3N$@NCF | 6 M KOH+0.2 M $Zn(Ac)_2$ | 1.04 | 51.4 | [2] |
| 3 | FeS/$Fe_3C$@Fe-N-C | 6 M KOH | 0.94 | 55.4 | [3] |
| 4 | $FeN_{0.0324}$@NiN4/C | 6 M KOH/0.2 M $Zn(OAc)_2$ | 0.73 | 62.8 | [4] |
| 5 | Co-MOF-74-HATP | 6 M KOH/0.2 M $Zn(OAc)_2$ | 0.93 | 56.6 | [5] |
| 6 | Co@C-CoNC | 6 M KOH/0.2 M $Zn(OAc)_2$ | 0.84 | 60.3 | [6] |
| 7 | CCSO/NC-2 | 6 M KOH/0.2 M $Zn(OAc)_2$ | 1.19 | 46.2 | [7] |
| 8 | NiFeVS | 6 M KOH/0.2 M $Zn(OAc)_2$ | 1.16 | 45 | [8] |
| 9 | CoSANi-N-CNT/CNF | 6 M KOH/0.2 M $Zn(OAc)_2$ | 0.89 | 54.1 | [9] |
| 10 | PAM-SC | 6 M KOH/0.2 M $Zn(OAc)_2$ | 0.9 | 56.7 | [10] |
| 11 | FeCoNiMoW | 6 M KOH/0.2 M $Zn(OAc)_2$ | 1.12 | 49.3 | [11] |
| 12 | FePc-BBL COF | 6 M KOH/0.2 M $Zn(OAc)_2$ | 1.04 | 52.9 | [12] |
| 13 | FeCo/N-CNT | 6 M KOH/0.2 M $Zn(OAc)_2$ | 0.822 | 58.5 | [13] |
| 14 | Ni3Fe13 DAC/N-GNs | 6 M KOH/0.2 M $Zn(OAc)_2$ | 1.24 | 41.2 | [14] |
| 15 | Pd55Au45 | 6 M KOH/0.2 M $Zn(OAc)_2$ | 0.92 | 52.8 | [15] |

## References


1. Zhang, W.J.; Dong, Q.; Zhao, Y.X.; Yang, W.Z.; Chen, J.Y.; Xi, F.C.; Li, Y.W.; Liu, X.X.; Zhou, J. BODIPY-based supramolecular complexes as oxygen reduction reaction and hydrogen evolution reaction bifunctional electrocatalysts for Zinc-air battery. *Chem. Eng. J.* **2025**, 507, 160369.
2. Meng, X.; Gao, S.; Liu, N.; Wu, P.; Fang, Z. Regulating N-doped biochar with Fe-Mo heterojunctions as cathode in long-life zinc-air battery. *Chem. Eng. J.* **2024**, 500, 157463.
3. Sun, S.; Yang, F.; Zhang, X.; Qian, J.; Wei, K.; An, J.; Sun, Y.; Wang, S.; Li, X.; Li, Y. Highly dispersed carbon-encapsulated FeS/$Fe_3C$ nanoparticles distributed in Fe-N-C for enhanced oxygen electrocatalysis and Zn-air batteries. *Chem. Eng. J.* **2024**, 487, 150673.
4. Liu, Q.; Chen, J.; Cao, L.; Wang, Y.; Qi, Y.; Wei, Y.; Ma, Q.; Huang, J.; Fan, X.; Feng, Y. Electronic Communication Between Single Atomic Nickel and Iron-Nitrogen Species Promote the Bifunctional Oxygen Evolution and Reduction for Efficient Rechargeable Zinc-Air Battery. *Adv. Funct. Mater.* **2025**, 35, 2424597.
5. Liu, W.; Liang, Y.; Huo, M.; Ma, N.; Qin, K.; Chang, J.; Xing, Z. Ligand engineering of Co-MOF-74 with hexaaminotriphenylene for enhanced oxygen reduction reaction in zinc-air batteries. *Nano Res.* **2025**, 18, 94907195.
6. Chandrasekaran, S.; Hu, R.; Yao, L.; Sui, L.; Liu, Y.; Abdelkader, A.; Li, Y.; Ren, X.; Deng, L. Mutual Self-Regulation of d-Electrons of Single Atoms and Adjacent Nanoparticles for Bifunctional Oxygen Electrocatalysis and Rechargeable Zinc-Air Batteries. *Nano-Micro Lett.* **2023**, 15, 48.
7. Cai, J.; Zhang, H.; Zhang, L.; Xiong, Y.; Ouyang, T.; Liu, Z.Q. Hetero-Anionic Structure Activated Co S Bonds Promote Oxygen Electrocatalytic Activity for High-Efficiency Zinc-Air Batteries. *Adv. Mater.* **2023**, 35, e2303488.
8. Sari, F.N.I.; Lai, Y.C.; Huang, Y.J.; Wei, X.Y.; Pourzolfaghar, H.; Chang, Y.H.; Ghufron, M.; Li, Y.Y.; Su, Y.H.; Clemens, O.; Ting, J.M. Electronic Structure Engineering in NiFe Sulfide via A Third Metal Doping as Efficient Bifunctional OER/ORR Electrocatalyst for Rechargeable Zinc-Air Battery. *Adv. Funct. Mater.* **2024**, 34, 2310181.

9. Poudel, M.B.; Balanay, M.P.; Lohani, P.C.; Sekar, K.; Yoo, D.J. Atomic Engineering of 3D Self-Supported Bifunctional Oxygen Electrodes for Rechargeable Zinc-Air Batteries and Fuel Cell Applications. *Adv. Energy Mater.* **2024**, 14, 2400347.
10. Jiao, M.; Dai, L.; Ren, H.R.; Zhang, M.; Xiao, X.; Wang, B.; Yang, J.; Liu, B.; Zhou, G.; Cheng, H.M. A Polarized Gel Electrolyte for Wide-Temperature Flexible Zinc-Air Batteries. *Angew. Chem. Int. Ed.* **2023**, 62, e202301114.
11. He, R.; Yang, L.; Zhang, Y.; Jiang, D.; Lee, S.; Horta, S.; Liang, Z.; Lu, X.; Ostovari Moghaddam, A.; Li, J.; Ibanez, M.; Xu, Y.; Zhou, Y.; Cabot, A. A 3d-4d-5d High Entropy Alloy as a Bifunctional Oxygen Catalyst for Robust Aqueous Zinc-Air Batteries. *Adv. Mater.* **2023**, 35, e2303719.
12. Zhang, Z.; Wang, W.; Wang, X.; Zhang, L.; Cheng, C.; Liu, X. Ladder-type π-conjugated metallophthalocyanine covalent organic frameworks with boosted oxygen reduction reaction activity and durability for zinc-air batteries. *Chem. Eng. J.* **2022**, 435, 133872.
13. Song, Y.; Li, W.; Zhang, K.; Han, C.; Pan, A. Progress on Bifunctional Carbon-Based Electrocatalysts for Rechargeable Zinc-Air Batteries Based on Voltage Difference Performance. *Adv. Energy Mater.* **2024**, 14, 2303352.
14. Dong, J.; Xie, X.; You, G.; Pan, Z.; Zhang, C.; Zhang, J. Highly dense graphene-supported NiFe dual-atom oxygen electrocatalysts with boosted bifunctional electrocatalytic performance for rechargeable zinc-air batteries. *Chem. Eng. Sci.* **2026**, 324, 123333.
15. Qiao, S.; Shou, H.; Xu, W.; Cao, Y.; Zhou, Y.; Wang, Z.; Wu, X.; He, Q.; Song, L. Regulating and identifying the structures of PdAu alloys with splendid oxygen reduction activity for rechargeable zinc-air batteries. *Energy Environ. Sci.* **2023**, 16, 5842–5851.
